# Night-Time Bracing Can Reduce Pain in Adults with Scoliosis: Six-Month Results of a Retrospective Controlled Study

**DOI:** 10.3390/jcm14134493

**Published:** 2025-06-25

**Authors:** Fabio Zaina, Martina Poggio, Sabrina Donzelli, René Castelein, Francesca Di Felice, Stefano Negrini

**Affiliations:** 1 ISICO (Italian Scientific Spine Institute), 20141 Milan, Italy; martina.poggio@isico.it (M.P.); francesca.difelice@isico.it (F.D.F.); 2 Istituto Auxologico IRCCS, 20145 Milan, Italy; sab.donzelli@gmail.com; 3 Utrecht University, 80145 Utrecht, The Netherlands; r.m.castelein-3@umcutrecht.nl; 4 Department of Biomedical, Surgical and Dental Sciences, University “La Statale”, 20122 Milan, Italy; stefano.negrini@unimi.it; 5 IRCCS Istituto Ortopedico Galeazzi, 20157 Milan, Italy

**Keywords:** scoliosis, chronic low back pain, brace

## Abstract

**Background:** Severe scoliosis can lead to chronic low back pain (cLBP) and may progress in adulthood. While day-time bracing is commonly used to alleviate pain and improve function, the role of night-time bracing remains unclear. This study aimed to assess the six-month effectiveness of a custom-made night-time brace in reducing pain in adults with scoliosis, compared to a prefabricated brace worn for 2–4 h during the day. **Methods**: A retrospective cohort study was conducted at a tertiary outpatient clinic specializing in spinal deformities. Adults with scoliosis (≥30° Cobb) and cLBP were divided into two groups: the study group used a custom-made night-time thoracolumbosacral orthosis (TLSO), while the control group wore a prefabricated brace (Peak) for 2–4 h daily. Pain and functional outcomes were assessed at baseline and after six months. **Results**: The study group included 25 women (mean age, 62.3 ± 9.5 years; Cobb angle, 60.4 ± 17.7°) who wore the night-time brace for an average of 7.2 ± 2.2 h per night. The control group comprised 20 women (mean age, 67.8 ± 10.5 years; Cobb angle, 61.9 ± 12.6°). At six months, the worst pain significantly improved in the TLSO group compared to the Peak group (F = 6.32, *p* = 0.0158). However, no statistically significant differences were observed between groups for back pain, leg pain, Core Outcome Measures Index (COMI), or Oswestry Disability Index (ODI). **Conclusions**: Night-time bracing shows interesting results on pain at six months in adults with severe scoliosis and back pain. These preliminary results open a new perspective that needs further verification and will help design more robust studies to verify what we found and identify the population more responsive to this approach.

## 1. Introduction

Scoliosis is a three-dimensional deformity affecting the spine. Its prevalence increases from 2–3% in adolescence to over 68% in subjects over 60 [1,2]. This increase comes from the addition of degenerative de novo scoliosis cases to those that originated during growth [3]. The degenerative cases are predominantly lumbar or thoracolumbar, while idiopathic cases can be thoracic or double curves. Despite these differences in localization, both types can hurt the quality of life (QoL) due to increased risk of back pain and progression of trunk imbalance [4]. The natural history of these types is slightly different, with a faster progression of degenerative types. The potential negative impact of both types on quality of life (QoL) and trunk balance is well documented [5].

Despite the current level of evidence, many patients are seeking conservative treatment. Similarly to adolescents with idiopathic scoliosis, the main pillars of this treatment for adults are bracing and physiotherapy scoliosis-specific exercises (PSSEs) [1]. After decades of traditional implementation, some papers have finally appeared. For bracing, a systematic review reported various designs, indications, dosages, and outcomes [6]. Usually, braces, prefabricated or custom-made, are used at variable dosages during the day. Patients need support during everyday activities to relieve pain and halt curve progression.

Recently, based on casual reports of a patient from our institute, we started using custom-made night-time bracing. This strategy could avoid a negative impact on muscles, significantly positively affecting pain and quality of life. Since there is only anecdotal data available, we designed this retrospective pilot study to verify if a custom-made brace worn during the night could help adult patients with chronic low back pain (cLBP) secondary to scoliosis on pain and quality of life in the short term. We also compared the results to a historical cohort from the same clinical prospective database treated with a prefabricated brace worn during the day [7].

## 2. Materials and Methods

### 2.1. Design

We designed a retrospective cohort study with a historical control group.

### 2.2. Participants

We searched our prospective database from February 2020 onward, when we started applying night-time custom-made rigid braces, to present time.

We included adults with idiopathic or degenerative scoliosis with a 30° Cobb angle or more and chronic low back pain who started wearing a custom-made night-time brace to improve pain. We included patients who had the brace’s prescription at our institution’s first evaluation and those already under treatment with PSSE in case they had stable or worsened pain.

Exclusion criteria: Secondary scoliosis, surgically treated patients, incomplete data (pre–post-treatment quality of life questionnaires, baseline radiographs).

We compared the results to a historical cohort from the same clinical prospective database treated with a prefabricated brace worn during the day [7].

### 2.3. Outcome Measures

We focused on pain as a primary outcome and disability as a secondary one. For pain, we applied the graphical rating scale (GRS) for back pain, leg pain, and worst pain (higher level of pain between leg and back). For the quality of life, we used the Oswestry Disability Index (ODI) and the Core Outcome Measure Index (COMI) [8].

### 2.4. Sample Size Calculations

The primary outcome measure for the power calculation is the variation in pain at the graphical rating scale (GRS) for worst pain (higher level of pain between leg and back as measured using the COMI) from baseline to the six-month follow-up. We assumed the standard deviation for the change to be 1.88. To be able to detect a difference of 2.0 points on the GRS between treatments (six months), we needed 14 subjects in both the active and control treatment groups at a power of 80% and with a two-tailed significance level < 0.05. Since the historical control group was made up of 20 subjects, we wanted at least to match this number. Sample size calculation is not commonly used in similar designs. Nevertheless, we performed it to be aware of the possibility of detecting even smaller differences.

### 2.5. Protocol

**Figure 1:**
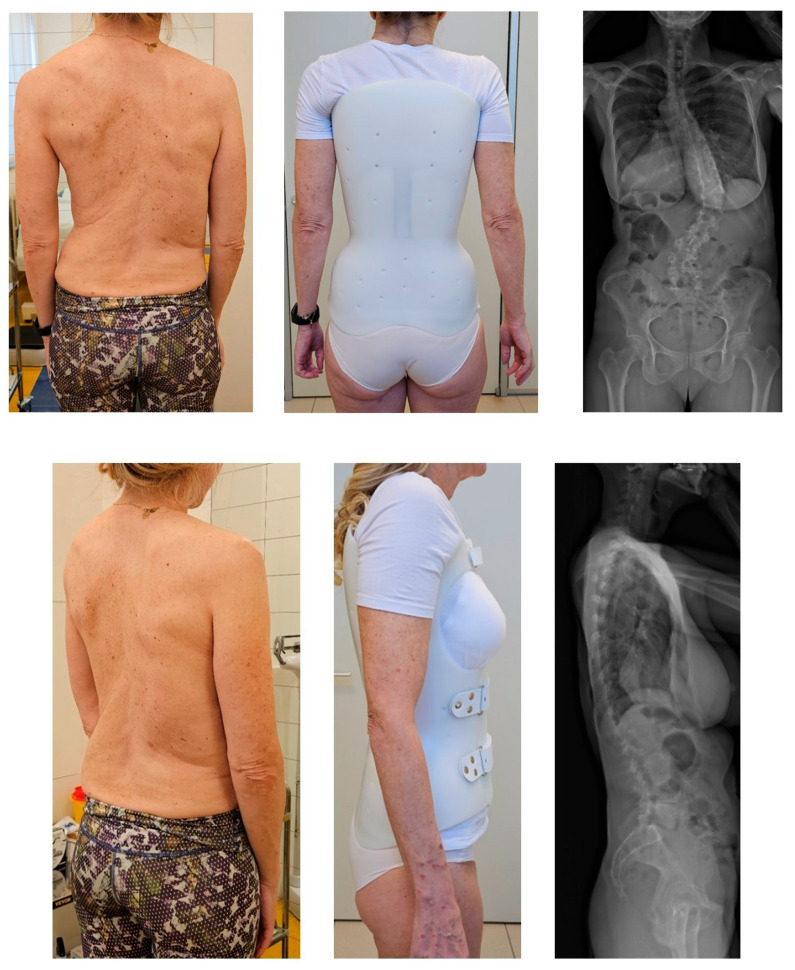
A patient at baseline, wearing a custom-made brace and her x-ray. Frontal (**upper** part) and lateral (**bottom**) view. On the left side, the trunk appearance at baseline. In the middle, the brace on the day it was delivered to the patient and checked by the treating physician and the orthotist. On the right, the most recent out-of-brace X-ray (EOS System) taken one month before the brace was built and delivered.

We compared the baseline evaluation made before the start of the brace treatment with the one made after 6 months of treatment with the brace. We also compared the results of the study group with those of an already published cohort of 20 patients treated with a prefabricated brace for 2–4 h during the day [7,9]. The dosage recommended was night-time, and the assessment was self-reported. All patients that were included received a custom-made 3 or 4 mm polyethene brace built using CAD-CAM technology. According to the current brace classification, the brace was a TLSO and rigid, had frontal and sagittal action, and was monocot with ventral closure [10]. We designed the brace to support the trunk and spine, avoiding any attempt to correct the deformity, trying to make them effective for pain and comfort (Figure 1). An expert physician checked the braces with an orthotist to optimize the effect and comfort. Since we were not seeking correction, we did not prescribe in-brace radiographs.

### 2.6. Statistical Analysis

The data was described according to the type of variables; we checked the distribution of questionnaire answers before defining the statistical tests needed. The skewness and Kurtosis resulted to be withing the range to consider the distribution normal.

To compare differences between brace treatments and changes over time, we used two-way ANOVA.

We also checked the results according to the minimal clinically important differences (MCIDs), which were 10 points at the ODI and 2 points at the COMI and GRS [7].

We defined improvement as a change equal or greater than the MCID, and we checked the association between the proportion of clinically meaningful improved patients and the brace type with chi2. We ran a logistic regression model to see if the OR of the obtained results is higher with one of the braces used to treat the patients.

### 2.7. Ethical Committee Approval

The local Ethics Committee Milano Area 2 approved this study (parere 453_2022). We respected the principles and indications of the Helsinki Declaration, and all included patients provided written informed consent.

## 3. Results

### 3.1. Population

Night-time bracing (TLSO group): Out of 32 consecutive patients who were treated at our institute, we included 25 women, with an average age of 62.3 years (±9.5) and an average spinal curvature of 60.4° Cobb (±17.7°). They wore the brace for an average of 7.2 h per day (±2.2 h) (Table 1). Seven were excluded because they had incomplete data (five had no baseline questionnaires, two no post-treatment questionnaires). We were unable to compare the data of the excluded patients because the outcome measures were missing.

Day-time Prefabricated Brace (Peak group): This group included 20 women, with an average age of 67.8 years (±10.5) and an average spinal curvature of 62° Cobb (±13°). They wore the brace for 2 to 4 h daily.

### 3.2. Pain Improvement

**Figure 2:**
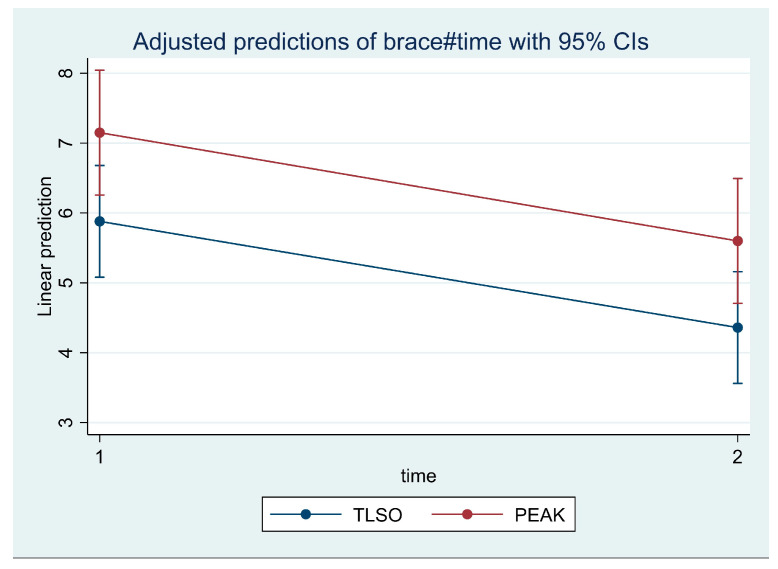
Changes in worst pain.

Both groups experienced similar improvements in back and leg pain, as shown in Table 2. However, after adjusting for time and repeated measures in the two-way ANOVA, we found there was a statistically significant improvement in the worst pain scores for patients in the TLSO group compared to those in the Peak group (F = 6.32, *p* = 0.0158). This improvement was also significant over time from the start to the end of the study (F = 20.54, *p* = 0.000) (Figure 2).

A minimal MCID was achieved in 44% of cases in the TLSO group and 30% in the day-time brace group, indicating no significant difference between the groups (chi^2^ = 0.93, *p* = 0.34).

Leg Pain: In total, 8% of patients in the TLSO group reached the MCID, compared to 15% in the Peak group (chi^2^ = 0.55, *p* = 0.46).

Back Pain: In total, 4% of patients in the TLSO group reached the MCID, compared to 20% in the Peak group (chi^2^ = 2.88, *p* = 0.09).

The proportion of subjects with results exceeding the MCID did not show a statistically significant difference between the two braces.

### 3.3. Logistic Regression Analysis

**Figure 3:**
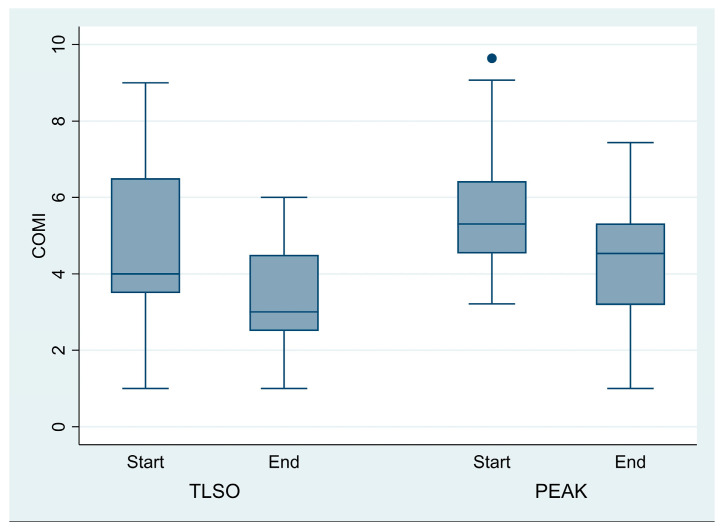
There is no significant effect of using the TLSO brace compared to the Peak group over time for the COMI score.

The analysis indicated that the odds of improvement were not different between the two braces. The COMI total results were 55% higher in patients treated with TLSO (OR = 0.55, *p* = 0.34, 95% CI: 0.2–1.9). The ODI results were 29% higher in patients treated with TLSO (OR = 0.71, *p* = 0.61, 95% CI: 0.2–2.6) (Figure 3).

**Figure 4:**
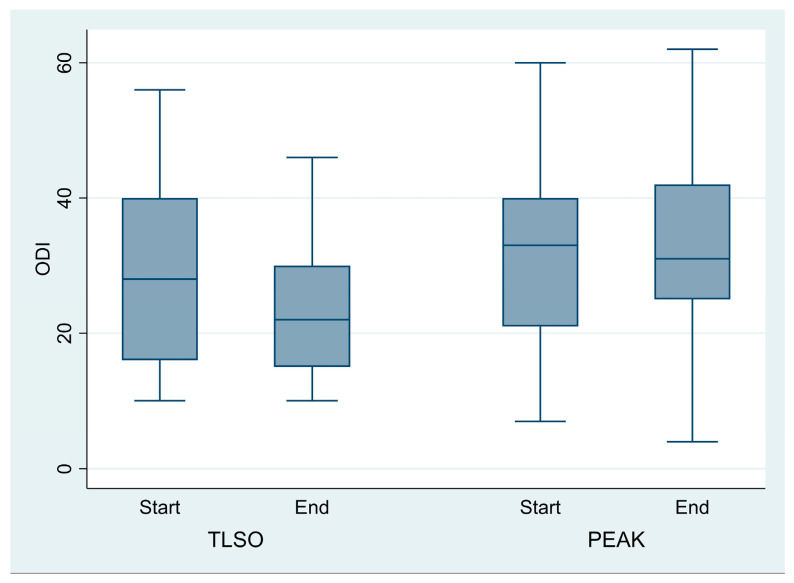
There is no significant effect of brace or time on ODI score.

Oswestry Disability Index (ODI):

The ODI measures disability related to lower back pain. In considering the Minimal Clinically Important Difference (MCID) for the ODI, 32% of patients treated with the TLSO showed improvement, compared to 25% in the day-time brace group. This difference was not statistically significant (chi^2^ = 0.26, *p* = 0.607) (Figure 4).

## 4. Discussion

The main findings of this study are the positive effects of custom-made braces on pain in patients with scoliosis and chronic pain. The results are similar to those of a prefabricated brace worn 2–4 h during the day [7].

Pain and disability are a common problem in adult patients with scoliosis [11], even if it is not clear if the prevalence and intensity of pain are higher than in the general population. The Iowa study, reporting a long-term follow-up of untreated scoliosis, showed similar pain duration and intensity for scoliosis than for the general population. However, it does not include a real control group of non-scoliosis [12]. Several papers, instead, have reported a higher prevalence and higher intensity of pain and disability in adult patients with scoliosis [13,14]. In this context, treatments should aim to stop or prevent the deformity’s structural degeneration and maintain or improve pain and quality of life. The main options to help patients with such a condition are exercises, bracing, and surgery, each presenting advantages and limitations [1]. Surgery is considered the most effective approach in severe cases [15]. Its efficacy is documented, and so are the risks connected to this procedure: the side effects and complication rates are high, especially in the elderly [16]. Moreover, not all patients are willing to be operated on, and it is unclear when this approach is preferable to conservative treatment [17]. There are not many papers comparing surgery and conservative. Moreover, the lack of a clear description of conservative treatment for scoliosis in almost all research comparing surgery to rehabilitation prevents any reliable comparison and recommendation of the best approach for individual patients.

Exercises are frequently proposed and eventually effective for pain and disability, but not all patients are compliant [18]. They need time to provide their results on symptoms, and sometimes they are less effective than patients expect. After the excellent results demonstrated in adolescents [19], bracing must also prove its role in adult treatment.

Despite being spread across many countries, only preliminary results are currently available, and the body of evidence needs improvement [6]. Evidence about bracing for pain and disability is largely based on low-quality studies, like case reports and non-controlled retrospective cohort studies [20,21,22]. A case report reported pain improvement at 10 days lasting 8 weeks [23]. A prospective study reported the positive results of a custom-made brace on pain and disability with a custom-made brace [24]. The authors selected only the positive responders through a test before providing the brace [24].

The largest study on bracing for adults is a retrospective cohort study including 739 patients [25]. The only inclusion criterium was the application of a rigid brace, so the population is a mix of different diagnoses, including post-surgical patients, camptocormia, thoracolumbar kyphosis, and Disabling Pain [25]. Originally, the authors referred to even more clinical diagnoses, but some were grouped as the mean age and average Cobb angle showed no significant difference. For instance, discopathy, lumbar instability, dysfunction, and a herniated disk were put in the same diagnostic group, and the group “disabling pain” included sciatica, neuropathic pain, rheumatic rigidity, and disability [25]. An objective measurement of pain and disability and the changes due to the brace treatment are missing [25].

A prospective study on a prefabricated brace recently showed interesting short-term results [9], persisting at six-month follow-up [7]. Their results were similar to those of other custom-made braces. The limit of such an approach is basically due to the reluctance of some patients, especially the youngest, to wear a brace during the day and the potential negative effect on spinal muscle strength. We started applying a different approach based on custom-made night-time bracing in clinical practice to overcome these challenges. The idea came by chance since a patient already performing exercises with partial benefit on pain wanted to try a different tool and approach. She was not interested in a day-time brace but was open to trying it at night. Since there is evidence reporting the possibility of preventing scoliosis progression during growth with night-time bracing, we thought that we could achieve some positive results also in adults [26,27,28]. This approach was satisfactory for patients and pushed our team to collect data to understand its potential role.

The findings of this paper are positive and surprising. For the first time, we can document a positive effect on chronic pain in patients with severe scoliosis. This paper opens new perspectives for further studies to understand how, when, and how long to brace these patients. Night-time bracing showed some efficacy in halting scoliosis progression during growth but was never tested before on pain and disability in adults [29].

The fascinating point is that the results seem similar to those achieved wearing a prefabricated brace for 2–4 h daily and also custom-made braces [7,24]. If this is confirmed, we will face a new scenario. One major drawback of a day-time brace is compliance, which tends to be reduced over time. One retrospective study showed that, despite the positive improvements in disability, at six-month follow-up, only 7 out of 29 patients were still wearing the brace for more than 4 h [30]. So, a night-time protocol could be better tolerated.

Concerns exist that prolonged use of spinal orthosis may lead to trunk muscle weakness and, eventually, atrophy. Although current evidence based on low-quality studies does not support this idea, at least for Lumbo-Sacral Orthosis [31,32], we need more data about the potential impact of Thoraco-Lumbar-Sacral Orthosis. Whatever the case, the probability that a night-time brace could impact muscle strength is very low. Moreover, this approach was well accepted even by younger patients, which could explain the slightly younger age of the study group. It is unclear how the night-time brace can work for pain, but it is worth starting new, more robust studies to confirm these findings. How the night-time brace works in adults with scoliosis and back pain is unclear. We can report that patients tell us that they feel straighter when they wake up after sleeping in the brace, and this helps them during the day.

Another future research scenario is to verify if night-time bracing can avoid or stop the progression of the deformity, as it can happen for minor curves in children. A retrospective study with long-term follow-up showed that day-time bracing could prevent or at least slow down scoliosis progression during adulthood and could be a good comparison [33].

This paper has several limitations. First of all, it has a retrospective design and small sample size. Nevertheless, this is the most cost-effective design for the preliminary testing of a new hypothesis before starting more expensive studies. Moreover, a preliminary study like this is mandatory to collect the data needed for the sample-size calculation for future research based on larger samples.

Another possible limit is the use of a historical control group. At baseline, despite having similar values of pain and disability, the two groups showed some differences, with the patients treated with the prefabricated brace being older than those treated with custom-made night-time ones and with more severe curves. Nevertheless, it is interesting to note a similar trend in improvements, and this is the first controlled study on bracing for adults with scoliosis.

Some concerns could be raised about the duration of follow-up. We did not check the very short-term results at 1 month like in other studies, and this is a limit since braces seem able to provide fast pain relief. Six months is the average duration in many studies evaluating chronic back pain. Of course, a longer follow-up can reinforce the validity of the results, and we will try to check this group again at one year and more.

Another potential limit is a confounding factor like PSSE. Nevertheless, the patients already performing PSSE showed stable or worsened pain six months before they entered the study, so its impact on the results appears at least limited.

A potential concern about custom-made braces could be their cost. When we collected these data, the cost of such a brace in Italy was about EUR 730, which was recently increased to about EUR 1000. The Peak brace (Tri-Point System) costs about EUR 740 in Italy. Both braces can be provided by the National Health System for free for patients with severe scoliosis.

## 5. Conclusions

The methodological limitations of this study raise questions about the generalizability of these findings until new data is available. Based on these limitations, we think an approach based on night-time bracing could be discussed with patients honestly, highlighting the uncertainty of the results due to the novelty and the few preliminary data points. Nevertheless, patients experiencing pain who cannot be operated on for major risks, those not interested in the surgical approach, and those who have a reduced QoL and are not interested in a day-time brace could consider this protocol. Moreover, younger patients, who usually refuse a day-time brace, could be interested in trying this approach.

Night-time bracing shows interesting positive results on pain at six months in adults with severe scoliosis and back pain. These preliminary results open a new perspective on the conservative approach to symptomatic spinal deformities during adulthood that need further verification. Moreover, it will help design more robust studies to verify what we found and identify the population more responsive to this approach.

## Figures and Tables

**Table 1:** Characteristics of the custom brace group (TLSO).

Characteristics	
Age	62.3 ± 9.5
BMI	23.1 ± 3.8
Curve (°Cobb)	60.4 ± 17.7
Single/Double Curve	18 single (72%)
	7 double (28%)
Localization	9 thoracic (36%)
	8 thoraco-lumbar (32%)
	8 lumbar (32%)
Diagnosis	15 AIS (60%)
	3 JIS (12%)
	6 adult scoliosis (24%)
Exercises (Min/week)	117.9 ± 48
Previous exercises	12 yes (48%), from 7.3 ± 6.2 months
	13 no (52%)
Wearing hours	7.2 ± 2.2
Age	62.3 ± 9.5
BMI	23.1 ± 3.8
LL	44.0 ± 11.4
PI	52.2 ± 16.4
PT	19.2 ± 10.2
PI-LL	7.2 ± 12.6

**Table 2:** Baseline and six-month values of pain and disability. Comparisons were made intra- and intergroup. * identifies statistically significant values.

	TLSO Brace				Peak Brace			
	Baseline	6 Months	*p* Value Intragroup		Baseline	6 Months		*p* Value Intergroup Post-Treatment
	Mean/Median (SD/95%CI)	Mean/Median (SD/95%CI)		*p* Value Intergroup Pre-treatment	Mean/Median (SD/95%CI)	Mean/Median (SD/95%CI)	*p* Value Intragroup	
Worst Pain (back or leg)	5.9 ± 1.9	4. ± 2.5	0.01 *	0.09	7.15 ± 2.03	5.6 ± 2.13	0.007*	0.06
Back Pain	5.7 ± 1.9	3.6 ± 1.9	0.001 *	0.28	6.55 ± 2.37	5.25 ± 2.69	0.06	0.07
Leg Pain	4.9 ± 2.9	3.3 ± 3.4	0.22	0.01 *	5.65 ± 3.03	4.35 ± 2.66	0.04 *	0.01 *
COMI	4.2 ± 2.0	3.1 ± 1.3	0.19	0.04 *	5.67 (5.11–6.79)	4.18 (3.34–5.02)	0.002 *	0.11
ODI	28.7 ± 15.1	23.8 ± 10.8	0.06	0.51	33.00 (25.26–38.43)	33.05 (26.30–39.79)	0.96	0.04 *

## Data Availability

Data are available at https://doi.org/10.5281/zenodo.14979410.
